# HIV‐1 promonocytic and lymphoid cell lines: an *in vitro* model of *in vivo* mitochondrial and apoptotic lesion

**DOI:** 10.1111/jcmm.12985

**Published:** 2016-10-18

**Authors:** Constanza Morén, Ingrid González‐Casacuberta, Carmen Álvarez‐Fernández, Maria Bañó, Marc Catalán‐Garcia, Mariona Guitart‐Mampel, Diana Luz Juárez‐Flores, Ester Tobías, José Milisenda, Francesc Cardellach, Josep Maria Gatell, Sonsoles Sánchez‐Palomino, Glòria Garrabou

**Affiliations:** ^1^ Muscle Research and Mitochondrial Function LaboratoryCellex‐IDIBAPSFaculty of Medicine‐University of BarcelonaInternal Medicine Department‐Hospital Clínic of Barcelona (HCB)BarcelonaSpain; ^2^ Centro de Investigación Biomédica en Red (CIBER) de Enfermedades Raras (CIBERER)MadridSpain; ^3^ Cellex‐IDIBAPSFaculty of Medicine‐University of BarcelonaInfectious Diseases Unit‐Hospital Clínic of Barcelona (HCB)BarcelonaSpain

**Keywords:** apoptosis, cell models, HIV‐infection, HIV progression, *in vitro* modelling, mitochondria

## Abstract

To characterize mitochondrial/apoptotic parameters in chronically human immunodeficiency virus (HIV‐1)‐infected promonocytic and lymphoid cells which could be further used as therapeutic targets to test pro‐mitochondrial or anti‐apoptotic strategies as *in vitro* cell platforms to deal with HIV‐infection. Mitochondrial/apoptotic parameters of U1 promonocytic and ACH2 lymphoid cell lines were compared to those of their uninfected U937 and CEM counterparts. Mitochondrial DNA (mtDNA) was quantified by rt‐PCR while mitochondrial complex IV (CIV) function was measured by spectrophotometry. Mitochondrial‐nuclear encoded subunits II–IV of cytochrome‐c‐oxidase (COXII‐COXIV), respectively, as well as mitochondrial apoptotic events [voltage‐dependent‐anion‐channel‐1(VDAC‐1)‐content and caspase‐9 levels] were quantified by western blot, with mitochondrial mass being assessed by spectrophotometry (citrate synthase) and flow cytometry (mitotracker green assay). Mitochondrial membrane potential (JC1‐assay) and advanced apoptotic/necrotic events (AnexinV/propidium iodide) were measured by flow cytometry. Significant mtDNA depletion spanning 57.67% (*P* < 0.01) was found in the U1 promonocytic cells further reflected by a significant 77.43% decrease of mitochondrial CIV activity (*P* < 0.01). These changes were not significant for the ACH2 lymphoid cell line. COXII and COXIV subunits as well as VDAC‐1 and caspase‐9 content were sharply decreased in both chronic HIV‐1‐infected promonocytic and lymphoid cell lines (<0.005 in most cases). In addition, U1 and ACH2 cells showed a trend (moderate in case of ACH2), albeit not significant, to lower levels of depolarized mitochondrial membranes. The present *in vitro* lymphoid and especially promonocytic HIV model show marked mitochondrial lesion but apoptotic resistance phenotype that has been only partially demonstrated in patients. This model may provide a platform for the characterization of HIV‐chronicity, to test novel therapeutic options or to study HIV reservoirs.

## Introduction

Data regarding the potential role of mitochondria in the cytopathogenicity of human immunodeficiency virus (HIV) have led to great interest in the study of the relationships between these two entities 1. Mitochondria have been closely linked to HIV infection, as a target of the deleterious effects of both HIV 2 and antiretroviral therapy (ART) 3 in relation to their involvement in the development of apoptosis. Mitochondrial and apoptotic alterations have been widely described in both naïve and treated patients *in vivo* 4, 5.

Human immunodeficiency virus has tropism for hematopoietic cells 6. This lentivirus is able to infect either lymphocytes or monocytes and macrophages, but the latter cells do not seem to undergo apoptosis following infection and represent a potential viral reservoir 7. Mitochondrial and apoptotic lesions in both lymphocytes and monocytes have been associated with accelerated HIV progression in naïve patients 8, 9 and in peripheral tissues from treated patients as a result of secondary effects of medication 10. A positive correlation has been described between apoptosis in peripheral blood lymphocytes and monocytes and the severity of HIV infection 11.

Whether mitochondrial and apoptotic malfunctions are causal factors rather than a consequence of differential HIV progression patterns remains to be elucidated. It is still unknown whether adequate mitochondrial function contributes to preventing mitochondrial‐driven apoptosis of the defence cells, increasing the organism defences against a specific viral antigen. It would be conceivable that correct mitochondrial function protects defence cells to undergo apoptosis, which, in turn, would slow down the infective capacity of HIV progression.

The interaction between mitochondria, apoptosis and HIV has been well‐described in HIV‐infected patients 12, 13 and several groups including ours, have assessed the association between mitochondrial and apoptosis alterations and the severity of HIV infection 8, 9, 14. Nevertheless, there is very little information on adequate mitochondrial characterization of *in vitro* models of HIV infection. Different animal models such as primate models have been used to further explore the disease and/or its treatment 15. However, high ratios of cost/effectiveness requiring complex facilities have been associated with these models. Cell models could contribute to solve these disadvantages. In this study, we used two different types of cell models which represent the main target cells for HIV (monocytes and lymphocytes). Human immunodeficiency virus‐1‐infected U1 and ACH2 cell lines are, respectively, promonocytic and lymphoid cell lines derived from uninfected U937 (same U1 cell line but uninfected) and CEM (same ACH2 cell line, but uninfected) precursor cells. U1 and ACH2 lines are characterized by harbouring one and two stable integrated copies of the HIV‐1 genome which replicates at a low rate, comparable to slow progression of the infection in patients. In these cell lines, HIV‐1 latently auto‐replicates itself, constituting a worldwide model to study chronic HIV infection 16, 17. One previous study has shown apoptotic resistance involving modulation of the apoptotic mitochondrial pathway in persistently infected HIV‐1 cells 18, however, accurate characterization of mitochondrial mechanisms and mitochondrial‐derived apoptosis has yet to be performed in these models of chronically infected cells.

The hypothesis of this study was that the mitochondrial and apoptotic damage initiated by HIV infection may contribute to the persistence of infection chronicity and progression. As a proof‐of‐concept, we expected mitochondrial genetics, function, expression and apoptotic levels to be altered in both chronically HIV‐1‐infected promonocytic and lymphoid cell lines. These parameters were initially determined in the chronically HIV‐1‐infected promonocytic and lymphoid cell lines U1 and ACH2, being U937 and CEM non‐HIV‐infected cells used as *in vitro* cellular controls respectively. Mitochondrial and apoptotic involvement in the progression of HIV infection could lead to the use of putative mitochondrial or apoptotic therapeutic strategies to deal with the chronicity of HIV patients.

In depth characterization of the mitochondrial and apoptotic pathways in these cell models may lead to elucidate whether these *in vitro* models resemble *in vivo* alterations observed in patients, providing a platform to test potential targets (such as mitochondrial or apoptotic therapeutic targets) to fight HIV infection.

## Materials and methods

### Cell lines

Chronically infected HIV‐1 promonocytic (U1) and lymphoid (ACH2) are cloned cell lines derived by limiting dilution cloning of U937 or CEM cells surviving an acute infection with HIV‐1 (LAV‐1 strain) first generated by Folks *et al*. 19, 20.

U1 and ACH2 cell lines, as well as their non‐HIV‐infected counterparts U937 and CEM, respectively, were cultured at 37°C in a fully humidified atmosphere with 5% CO_2_ in RPMI‐1640 medium (BioWhittaker, LONZA Portsmouth, NH, USA) supplemented with 10% foetal calf serum, and 1% penicillin‐streptomycin, by trained personnel in P3 facility cores devoted to viral replication.

Mitochondrial and apoptotic parameters and experimental setups were simultaneously evaluated in these HIV‐1‐infected cell lines and their corresponding uninfected controls, and were run in parallel with both infected and control cell lines. Cell cultures were grown into 12 experiments (including four lines in parallel). Experimental measurements were run, at least, in triplicates.

### Mitochondrial DNA depletion through multiplex real time PCR

A mitochondrial DNA (mtDNA) depletion study was performed as described. Total DNA was phenol‐chlorophorm‐extracted, spectrophotometrically quantified and diluted at 5 ng/μl. Multiplex real‐time PCR (PCR Applied Biosystems (Foster City, CA, USA) 7500 Real Time PCR System) was performed with 96 round bottom well plates with the simultaneous determination of the mitochondrial 12S ribosomal RNA (mt12SrRNA) gene and the constitutive nuclear RNAseP gene (nRNAseP). The former used mtF805 (5′‐CCACGGGAAACAGCAGTGAT‐3′) and mtR927 (5′‐CTATTGACTTGGGTTAATCGTGTGA‐3′) with the TaqMan Probe 6FAM‐5′‐TGCCAGCCACCGCG‐3′‐MGB (Sigma‐Aldrich, St. Louis, MO, USA). The latter used a commercial kit (4304437; Applied Biosystems). Each well included 25 ng of total genomic DNA diluted in 20 μl total reaction mixture containing: 1× TaqMan Universal PCR Master Mix (ABI P/N 4304437), 1 μl RNAseP commercial kit and 125 nM of each mtDNA primer and 125 nM of mtDNA probe.

The PCR was set at 2 min. at 50°C, 10 min. at 95°C, followed by 40 cycles each of 15 sec. of denaturalization at 95°C and 60 sec. of annealing/extension at 60°C. The mt12SrRNA gene was normalized by determining the nRNAseP nuclear gene and expressed as mt12SrRNA/nRNAseP ratio.

### Mitochondrial function by enzymatic activities

Complex IV (CIV) enzymatic activity was measured as an experimental parameter 21 representative of mitochondrial function. The enzymatic activity of CIV was measured following national standardization rules of the Spanish network for the study of mitochondrial respiratory chain (MRC) enzyme activities. This enzymatic assay was spectrophotometrically measured at 37°C at a wavelength of 550 nm including an internal control of pig muscle sample with known reference values and expressed as nmols/min.mg protein.

### Protein subunits content and mitochondrial apoptotic events by western blot

Mitochondrial and nuclear DNA encoded subunits (COXII and COXIV, respectively) of CIV, in addition to voltage‐dependent anion chanel‐1 (VDAC‐1) and caspase‐9 content were analysed by western blot. In brief, 20–30 μg crude cell lysates were mixed 1:5 with a solution containing 50% glycerol, 10% SDS, 10% β‐mercapto‐ethanol, 0.5% bromophenol blue and 0.5 M Tris (pH 6.8), incubated at 99°C for 5 min. for protein denaturalization and electrophoresed on 0.1% SDS ranging from 7% to 13% of polyacrylamide gels. Proteins were transferred onto nitrocellulose membranes for 7 min. using an automatic system. Blots were probed with (*i*) a monoclonal antibody (moAb) recognizing the mtDNA‐encoded human COXII subunit as a marker of the mitochondrial protein synthesis rate (A6404; Molecular Probes, Eugene, OR, USA), (*ii*) a moAb elicited against the nuclear DNA encoded human COXIV subunit as a marker of the nuclear protein synthesis rate (A21347; Molecular Probes) and (*iii*) an anti‐β‐actin moAb (A5441; Sigma‐Aldrich) as a loading control of overall cell protein content. The protein subunits content was normalized by the content of β‐actin signal to establish the relative abundance per overall cell protein. Antimouse and anti‐rabbit secondary antibodies were used depending on the primary antibody 22. Mitochondrial and nuclear protein synthesis ratios were expressed, respectively, as COXII/β‐actin and COXIV/β‐actin.

Apoptosis approaches were measured by western blot (as aforementioned) using the moAb raised against VDAC‐1 (529536, antiporin 31HL; Calbiochem, Darmstadt, Alemania) as a marker of early apoptotic mitochondrial events and moAB against caspase‐9 (ab2324; Abcam, Cambridge, UK) as marker of advanced apoptotic mitochondrial events, normalized by β‐actin content and expressed as the VDAC‐1/β‐actin and caspase‐9/β‐actin ratios.

### Mitochondrial content quantified by citrate synthase enzymatic activity

Citrate synthase (CS) enzymatic activity was measured as a reliable marker of mitochondrial content 21. Enzymatic activity was spectrophotometrically measured following national standardization rules of the Spanish network for the study of MRC enzyme activities. This enzymatic assay was performed at 37°C including an internal control of pig muscle sample with known reference values and assessed at a wavelength of 412 nm and expressed as nmols/min.mg of protein.

### Mitochondrial content, mitochondrial depolarization and advanced apoptosis/necrosis by flow cytometry

Mitochondrial content was determined by mitotracker green (MTG), and mitochondrial membrane potential (MMP) was estimated by JC‐1 staining as reported elsewhere 23, 24 in cell lines either in the presence or absence of the mitochondrial pro‐apoptotic and depolarizing stimuli valinomycin. Advanced apoptosis and necrosis phenomena were assessed using the annexin V and propidium iodide (PI) ratio by means of flow cytometric analysis. Briefly, a total of 1 ml of complete culture media containing roughly 2 × 10^5^ cells was prepared for different reaction procedures and subjected to incubation: (*i*) in the absence of any dye used for the autofluorescence calculation, (*ii*) with 200 nM MTG fluorophore (M‐7514; Molecular Probes) for 30 min. for mitochondrial content quantification, (*iii*) with 0.02% JC1 dyer (T‐3168; Molecular Probes) for 10 min. for MMP assessment, (*iv*) with 0.02% JC1 fluorophore plus 0.05% valinomycin pro‐apoptotic and depolarizing stimuli reagent (60403; Sigma‐Aldrich) for 10 min. and (*v*) with 0.05% annexin V plus PI (556463; BD Biosciences, East Rutherford, NJ, USA) for 10 min. to assess advanced apoptotic and necrotic events. All cytometric analyses were performed in a FACScalibur cytometer with an argon ion laser tuned at 488 nm and a diode laser tuned at 635 nm (Becton Dickinson, San José, CA, USA). Results were expressed as median or percentage of cells with specific fluorescence.

### Statistics

Results were expressed as mean ± S.E.M. Descriptive statistics were performed using the Statistical Package for the Social Sciences (SPSS) software (IBM inc. SPSS Statistics Chicago, IL, USA). A single filter was applied to discard extreme values. Statistical analysis was performed with non‐parametric Kruskal–Wallis H and Mann–Whitney *U*‐tests, and the level of significance was considered at *P* < 0.05 (for a confidence interval of α = 95%).

## Results

**Figure 1:**
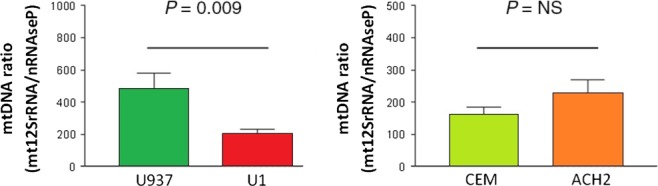
mtDNA content. This figure shows the mtDNA *versus* nuclear DNA ratio (expressed in arbitrary units) in the infected promonocytic U1 cell line with respect to its uninfected U937 control (left), *P* < 0.01, as well as in the HIV‐1‐infected lymphoid ACH2 cell line with respect to its CEM control (right), *P* = NS. MtDNA: mitochondrial DNA, Mt12SrRNA: mitochondrial 12SrRNA ribosomal gene, nRNAseP: nuclear RNAseP gene, NS: not significant.

Mitochondrial DNA content showed a significant decrease in the promonocytic latently infected HIV‐1 cell line U1 compared to its respective uninfected U937 control cell line (204.64 ± 28.07 *versus* 483.34 ± 97.61; *P* < 0.01). However, the lymphoid latently infected HIV‐1 cell line ACH2 showed non‐significant variations of mtDNA content compared to the uninfected CEM control cells (227.32 ± 42.99 *versus* 162.59 ± 22.35, *P* = NS; Fig. 1).

**Figure 2:**
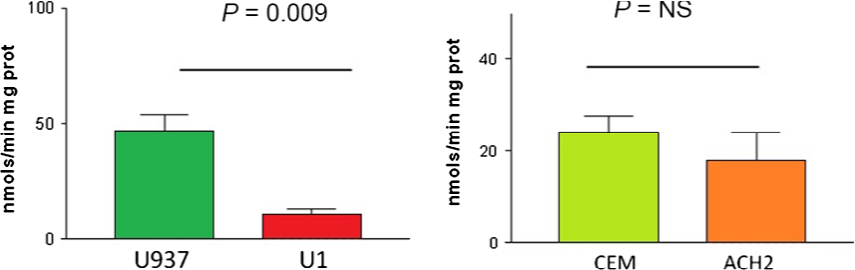
CIV enzymatic activity. This figure shows the enzymatic activity of complex IV of the mitochondrial respiratory chain of promonocytic (left) and lymphoid (right) cell lines expressed in nmols/min mg protein. A significant decrease in complex IV activity was observed in the infected promonocytic cell line U1 with respect to its uninfected U937 control, *P* < 0.01. NS: not significant.

Mitochondrial CIV enzymatic activity was then quantified. In agreement with the above results, CIV enzymatic activity significantly decreased in infected promonocytic U1 cells with respect to uninfected U937 control cells (10.57 ± 2.46 *versus* 46.84 ± 7.30, *P* < 0.01). On the other hand, this value remained non‐significantly altered, although showing a trend to decrease in the infected ACH2 lymphoid cell line compared to uninfected CEM control cells (17.86 ± 6.12 *versus* 24.16 ± 3.45, *P* = NS; Fig. 2).

**Figure 3:**
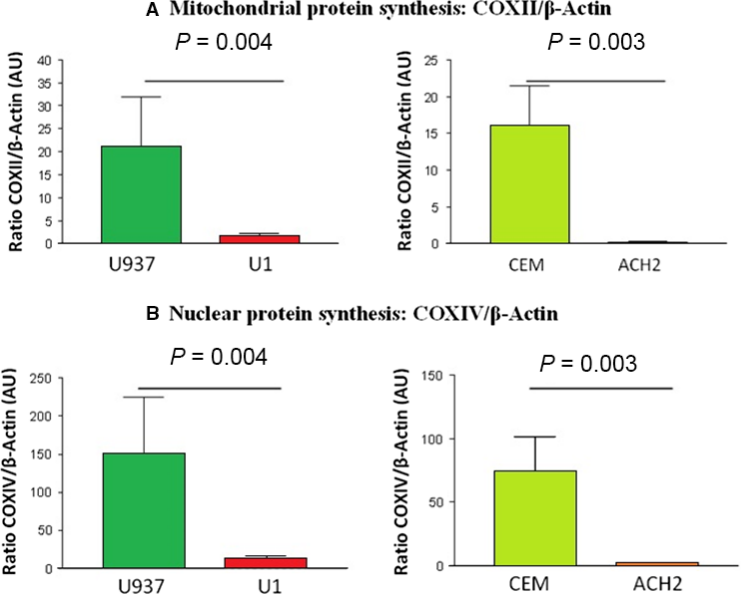
(**A**) COXII content. This figure shows COXII protein subunit content in arbitrary units with expression of the levels of mitochondrial DNA encoded subunit II of complex IV of promonocytic (left) and lymphoid (right) cell lines normalized by β‐actin content. A significant decrease in mitochondrial protein synthesis was observed in promonocytic and lymphoid U1 and ACH2 infected cell lines with respect to their uninfected U937 and CEM controls. COX: cytochrome c oxidase, AU: arbitrary units. (**B**) COXIV content. This figure shows COXIV protein subunit content in arbitrary units with expression of the levels of nuclear DNA encoded subunit IV of complex IV of promonocytic (left) and lymphoid (right) cell lines normalized by β‐actin content. A significant decrease in nuclear protein synthesis was observed in promonocytic and lymphoid U1 and ACH2 infected cell lines with respect to their uninfected U937 and CEM controls. COX: cytochrome c oxidase, AU: arbitrary units.

Thereafter, the content of mitochondrial protein subunits was assessed by the expression of both the mtDNA‐encoded COXII (Fig. 3A) and the nuclear encoded COXIV (Fig. 3B), components of the MRC CIV. The expression of these two subunits was reduced in both chronically HIV‐1‐infected cell lines studied compared to their respective uninfected control cell lines (U1 *versus* U937 and ACH2 *versus* CEM, *P* < 0.001 in all cases).

**Figure 4:**
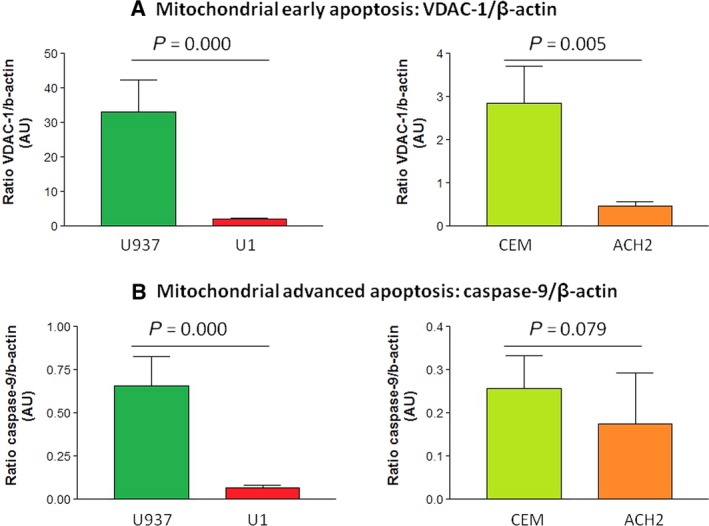
(**A**) VDAC‐1 content. This figure shows the VDAC‐1 content expressed in arbitrary units. The expression of the levels of VDAC‐1 content normalized by β‐actin of promonocytic (left) and lymphoid (right) cells. A significant decrease in early mitochondrial apoptosis was observed in promonocytic and lymphoid U1 and ACH2 infected cell lines with respect to their uninfected U937 and CEM controls. VDAC‐1: voltage‐dependent anion channel‐1, AU: arbitrary units. (**B**) Caspase‐9 content. This figure shows the caspase‐9 content expressed in arbitrary units. The expression of the levels of caspase‐9 content normalized by β‐actin of promonocytic (left) and lymphoid (right) cells. A decrease in advanced mitochondrial apoptosis was observed in promonocytic (significant) and lymphoid (non‐significant) U1 and ACH2 infected cell lines with respect to their uninfected U937 and CEM controls. AU: arbitrary units.

Moreover, early and advanced apoptotic mitochondrial events estimated as VDAC‐1 and caspase‐9 content, respectively, were significantly reduced in infected U1 and ACH2 infected cell lines *versus* their respective uninfected counterparts (U937 and CEM, respectively, *P* < 0.05 in all cases, except for caspase‐9 in lymphoid cell lines: *P* = 0.079; Fig. 4A and B).

**Figure 5:**
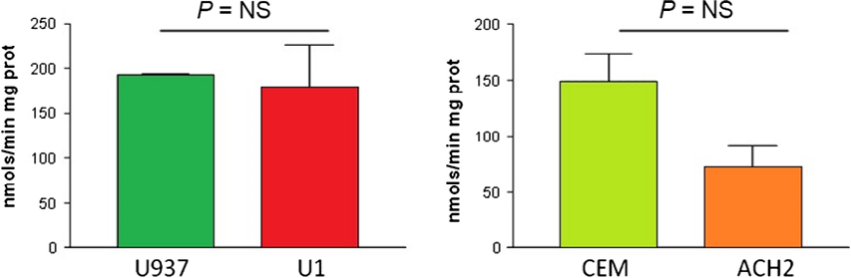
Mitochondrial content. This figure shows the mitochondrial content measured by citrate synthase enzymatic activity expressed in nmols/min mg protein of promonocytic (left) and lymphoid (right) cells. No significant changes were observed in infected promonocytic and lymphoid U1 and ACH2 cell lines with respect to their uninfected U937 and CEM controls. NS: not significant.

The mitochondrial content did not show any statistically significant differences, quantified by either CS activity (Fig. 5) or by the MTG assay (data not shown).

Finally, the comparisons with mitochondrial membrane depolarization in addition to advanced apoptosis and the necrosis markers assessed by flow cytometry did not reach statistical significance, although some parameters showed a bias. Thereby, the U1 promonocytic infected cell line showed a trend to lower levels of depolarized mitochondria than their uninfected U937 control cells, although these differences were not statistically significant (0.22 ± 0.16 *versus* 2.38 ± 2.38 respectively). This trend was slight in the HIV‐1‐infected ACH2 lymphoid cell line *versus* uninfected CEM cells (9.99 ± 9.74 *versus* 12.44 ± 10.85, *P* = NS). The formerly described increment pattern was also exhibited in promonocytic U1 infected cells *versus* U937 uninfected control when cells were exposed to a pro‐apoptotic reagent (valinomycin) (1.54 ± 1.54 *versus* 10.22 ± 10.22, respectively), but this exacerbation pattern was not observed in ACH2 lymphoid cells *versus* uninfected control CEM cells (13.72 ± 11.51 *versus* 12.91 ± 10.88 respectively).

The rate of cells of either promonocytic or lymphoid origin undergoing apoptosis or necrosis measured by annexin V plus PI did not render significant differences. A trend towards a decrease was observed in HIV‐1‐infected U1 promonocytic cells with respect to their U937 controls (9.92 *versus* 27.63 respectively), whereas an increase was observed in HIV‐1‐infected ACH2 lymphoid cells in comparison with their CEM control cells (23.40 *versus* 4.32).

## Discussion

In this study, we compared mitochondrial and apoptotic parameters in chronically HIV‐1‐infected promonocytic and lymphoid cell lines with their uninfected counterparts to evaluate whether they could be used as HIV infection models of the *in vivo* mitochondrial and apoptotic lesion characteristic of HIV‐infected patients. Most of the mitochondrial and apoptotic parameters were altered in the HIV‐infected cell lines, especially in the promonocytic lineage, resembling *in vivo* alterations in case of mitochondrial findings. However, contrarily to what is observed *in vivo*, both promonocytic and lymphoid HIV‐infected models showed resistance to undergo apoptosis. In the case of the promonocytic infected cell line U1, this may lead to a useful model to study HIV reservoirs, frequently established in these cell lineages. However, *in vivo* apoptosis of lymphocytes followed by infection was not observed in the ACH2 model, except for the trends of annexin V and PI measured by flow cytometry.

Mitochondrial and apoptotic abnormalities have been postulated to be the basis of collateral effects such as the development of hyperlactatemia, lipodystrophy and neuropathy associated with both HIV infection and its treatment 25, 26, 27, 28. Although most mitochondrial changes are considered to be the result of HIV apoptotic capacity and the secondary effects derived from ART 12, there is a growing evidence of the crucial role of mitochondria in the dynamics of HIV infection 29. Despite the interest in several areas of the HIV infection process such as HLA, polymorphisms in viral co‐receptors, antibodies, chemokines and defensins, among others 30, 31, the role of mitochondria has become outstanding in this process 29, 32.

The mitochondrial and apoptotic damage induced by HIV infection may exacerbate these deleterious effects in a kind of vicious cycle, terminating in the loss of cell defence capability of the host and the progression of infection. Studies performed to date have highlighted the need to establish an *in vitro* model to further investigate possible therapeutic approaches to revert mitochondrial or apoptotic damage and determine novel tools to fight HIV progression.

### Mitochondrial parameters

In our cell models, most of the mitochondrial parameters were affected in both cell lines. Mitochondrial genome content and functional enzymatic activity were sharply depleted in U1 promonocytic cells, without being significantly modified in the ACH2 lymphoid cells. Both infected cell lines exhibited a marked decrease in MRC subunit expression which, in case of the promonocytic cells, goes in agreement with the outstanding dysfunction of the MRC. The expression of mitochondrial protein subunits of MRC was above 90% decreased in most cases compared to their uninfected counterparts. These mitochondrial and nuclear encoded subunits were diminished in both HIV‐1‐infected cell lines, suggesting both mitochondrial and nuclear gene malfunctions in all cases.

### Apoptotic parameters

A link between HIV infection and apoptosis has been widely reported 33. Some viral proteins interact with mitochondrial targets, leading to apoptosis through different pathways 34, 35. This is the case of the viral protein R (Vpr), the HIV‐1‐trans‐activating protein (Tat) and the viral protease (Pr), which can directly interact with components of the mitochondrial permeability transition pore (Vpr interacts with the adenine nucleotide translocator ANT attached to VDAC, thus prompting mitochondrial depolarization) 32, translocate proapoptotic proteins into the mitochondria (Tat mediates Bim translocation) 33, activate caspases (Vpr activates caspases 3 and 9) 34 or inactivate anti‐apoptotic proteins (Vpr inactivates HAX‐1 and Pr inactivates Bcl‐2) 34, 35, ultimately causing cell death. Consequently, the marked significant decrease in the early and advanced apoptotic markers (VDAC‐1 and caspase‐9 proteins) in both infected cell lines studied was unexpected. These results are in agreement with the lower levels of early apoptotic events of depolarized mitochondrial membranes observed with JC1 by flow cytometry in both infected U1 and ACH2 cell lines, although such trends did not achieve statistical significance. Our data strongly indicate that latently infected cells are less susceptible to undergo apoptosis compared to uninfected cells. These results support a previous study 18 which was mainly focused on the reaction of specific cell lines against different exogenous pro‐apoptotic and stress‐induced stimuli. Although HIV infection most frequently leads to cell death, the latently HIV‐1‐infected cell lines studied only showed a reduced apoptotic behaviour which may be indicative of a viral strategy to survive 36. In this sense, both promonocytic and lymphoid HIV‐1‐infected cell lines showed different patterns in advanced apoptosis and necrosis phenomena tested by flow cytometry through annexin V plus PI staining. A trend to decrease in advanced apoptotic events was observed in the infected U1 promonocytic cell lines compared to their controls, whereas a tendency to increase was observed in infected ACH2 lymphoid cell lines. These data are also in agreement with the lower sensitivity to apoptosis previously observed in promonocytic *versus* lymphoid cells 37.

### Validation of both *in vitro* models

Considering all these results, our findings suggest that these cell models may at least partially reproduce *in vitro* the alterations observed *in vivo* in HIV‐infected patients 4, 8, 9, 12, 13 that should be further characterized in depth.

From a mitochondrial point of view, both promonocytic and lymphoid cell lines showed pronounced abnormalities with slightly different patterns. Previous studies reported different behaviour for promonocytic and as well as lymphoid cell lines 37 or monocytes/lymphocytes 36 suggesting that different molecular mechanisms may depend on cell types considered. In our study, although both promonocytic and lymphoid cell lines showed changes in MRC expression, only the promonocytic cells (not the lymphoid lineage) showed significant alterations at the mitochondrial genetic and functional levels. In this context, it was surprising to notice the significant reduced content of subunits COXII and COXIV in the lymphoid infected lines despite the trends towards increased levels of mtDNA and only slightly reduced enzymatic activity of the complex IV. It would be conceivable that, despite the decrease in the subunit protein levels is observed, the threshold to observe a defective functionality of the enzyme had not been yet reached, probably due to the homeostatic up‐regulation of mtDNA content in these cells. All these mechanisms were not observed in promonocytic infected cell lines, which showed decreased ratios in all genetic, functional and expression mitochondrial parameters fully resembling *in vivo* conditions.

From the apoptotic point of view, both infected cell lines showed a trend to a resistance to apoptotic events.

A previous study reported lower MMP in peripheral blood mononuclear cells from HIV patients, as well as higher apoptotic/necrotic events in these cells 35. In this study non‐significant trends were found in MMP. In addition, most of apoptotic/necrotic parameters measured (mitochondrial pore and caspase‐9 activation or phosphatidylserine expression and nuclear DNA fragmentation) yielded to similar results suggesting the resistance to undergo apoptosis of HIV‐infected cell models 18, 33 and, as opposite as *in vivo* conditions of blood cells not becoming reservoirs 4, 5.

Our findings point out the use of the present *in vitro* models as largely useful for mitochondrial monitoring. We encourage other researchers to explore in depth apoptotic events in this and other *in vitro* models. Importantly, our results clearly mimic the *in vivo* mitochondrial features of HIV infection, (especially in the promonocytic cell line), rather than the *in vivo* apoptotic conditions. Apoptotic resistance in infected lines might be derived from the genetic modification of the cancerous lineages that do not undergo apoptosis following viral genome insertion. Promonocytic apoptotic resistance may reflect the *in vivo* percentage of monocytes capable to constitute reservoirs and, subsequently, become more resistant to apoptosis. Resistance to apoptosis of ACH2 lymphoid cell line should be taken with caution when willing to reproduce *in vivo* pathophysiology of HIV infection. The observed findings, suggest that such alterations might have HIV clinical consequences as previously described *in vivo* 11 and point out to the involvement of mitochondrial status in HIV progression and chronicity. In addition, this study provides complementary data to the apoptosis approaches previously considered 15.

In spite of strained HIV‐1 replication in the infected cell lines used in this study, the assessment of the mitochondrial and apoptotic parameters investigated led to the description of significant changes compared to the respective uninfected control cell lines. It remains unknown whether these disturbances would be increased in activated HIV‐1‐infected cell lines (with the addition of HIV replication inducer factors).

A limitation of this study was the resistance to apoptosis of the cancerous infected cell lines, which remain viable regardless of HIV infection and would have died in natural conditions. Nonetheless, despite these limitations, due to the same cancerous origin of both control and infected lines, it is presumable that the significant changes found between cell lines are comparable and attributable to the infection itself. Thereby, we consider that the models studied may constitute adequate *in vitro* models to monitorize the patient dynamics at apoptotic and especially at mitochondrial level.

In summary, this study provides evidence that the mitochondrial and, in a lesser extent, apoptotic pathways are seriously affected in latently infected HIV‐1 promonocytic and lymphoid cells. The availability of an *in vitro* HIV model is essential for translational research. Studies using these cell models of HIV infection may lead to the development of novel therapeutic tools 38, including apoptotic and mitochondrial targets 39, as strategies to reverse the lesions exerted by the virus.

## Conflicts of interest

None of the authors has any financial, consultant, institutional and other relationship that might lead to bias or a conflict of interest for the present manuscript.
